# Biopesticide synergy when combining plant flavonoids and entomopathogenic baculovirus

**DOI:** 10.1038/s41598-020-63746-6

**Published:** 2020-04-22

**Authors:** William T. Hay, Robert W. Behle, Mark A. Berhow, Andie C. Miller, Gordon W. Selling

**Affiliations:** 1 Plant Polymer Research Unit, USDA, Agricultural Research Service, National Center for Agricultural Utilization Research, 1815 N, University Street, Peoria, IL 61604 USA; 2 Crop Bioprotection Research Unit, USDA, Agricultural Research Service, National Center for Agricultural Utilization Research, 1815 N, University Street, Peoria, IL 61604 USA; 3 Functional Foods Research Unit, USDA, Agricultural Research Service, National Center for Agricultural Utilization Research, 1815 N, University Street, Peoria, IL 61604 USA; 4 Bradley University, Department of Biology, 1501W. Bradley Ave Olin Hall 101, Peoria, IL 61625 USA

**Keywords:** Entomology, Plant sciences, Virology

## Abstract

Four crop plants known to be hosts for the lepidopteran *Trichoplusia ni* (soybean, green bean, cotton, and cabbage) were treated with the biopesticide AfMNPV baculovirus in a dosage response assay. Treated soybean had, on average, a 6-fold increase in virus activity compared with the other crops. Leaf trichomes on soybeans were not found to be responsible for the observed increase of insecticidal activity. Three flavonoid compounds (daidzein, genistein, and kaempferol) were uniquely found only in the soybean crop, and were not detected in cotton, cabbage, or green bean plant matter. The individual flavonoid compounds did not cause *T ni*. mortality in no-virus assays when incorporated into artificial insect diet. The combination of the three flavonoid compounds at leaf level concentrations significantly increased baculovirus activity in diet incorporation assays. When the daidzein, genistein, and kaempferol were added to artificial diet, at 3.5–6.5 × leaf level concentrations, virus activity increased 1.5, 2.3, and 4.2-fold for each respective flavonoid. The soybean flavonoid compounds were found to synergistically improve baculovirus activity against *T. ni*.

## Introduction

Biological control has been defined as the use of predators, parasites, or pathogens to manage crop pests in a highly specific and environmentally friendly method^1^. The development of microbial-based biopesticides represents the augmentation of biological control for insect pests of plants and is increasingly viable as a commercial control tactic. The use of naturally occurring biological control agents allows growers to precisely target a pest population^2^. Research on this topic often focuses on the interaction specifically between the pathogen and target insect, such as the control efficacy provided by the application of a biopesticide. However, plants primarily protect themselves through the production of chemical defense compounds, which may alter aspects of this pest/pathogen interaction^3^. In a natural forest ecosystem, plant chemistries contribute to complex interactions depending on many parameters of the particular ecosystem, such as the density of oak trees in a forest^4^. Although comparatively less complex, reports of interactions among crop, pest, and pathogen vary for monoculture cropping systems. The susceptibility of *Helicoverpa zea* to Helicoverpa zea nucleopolyhedrovirus was found to be greater when fed on soybean rather than cotton, however, no specific plant chemistry was identified^5^. In okra and tomato, induced plant defense chemistries are correlated with reduced baculovirus infection of the lepidopteron *Heliothis virescens* (Fabricius), however induced systemic acquired resistance in cotton foliage had no effect on baculovirus infection of the lepidopteron *Helicoverpa armigera* (Hübner)^6,7^.

A naturally occurring baculovirus was isolated and incorporated in an integrated pest management system in Brazil, which successfully controlled the soybean defoliating lepidopteran velvet bean caterpillar, *Anticarsia gemmatalis* Hübner^8,9^. Another baculovirus isolated from *Anagrapha falcifera* Kirby, (Anagrapha falcifera multi-nuclear polyhedrosis virus - AfMNPV) can also be used as a biological control of key lepidopteran pests^10^. During preliminary research on formulation development with AfMNPV, we observed that larval mortality of lepidopteran *Trichoplusia ni* Hübner was substantially and unexpectedly high on soybean leaves treated with only AfMNPV, as compared with larvae feeding on virus treated cotton, cabbage, and the closely related green bean. This observation prompted evaluations to determine whether differences in plant morphology or chemistry significantly interacted with baculovirus infection.

The objective of this investigation was to determine the cause of increased viral activity against *T. ni* when baculovirus was applied to soybeans by (1) determining the comparative AfMNPV activity against *T. ni* for treatments applied to soybean, cotton, cabbage, and green bean, (2) determine whether physical leaf structures such as trichomes (pubescence) affect insecticidal activity of the virus, (3) determine flavonoid composition of leaf tissues, (4) determine whether identified flavonoids affect AfMNPV activity against *T. ni* in flavonoid-diet assays.

## Materials and Methods

### Materials

Genistein (98%), kaempferol (99%), trifluroacetic acid (99%), and formic acid (98%) were purchased from Sigma, St. Louis, MO. Daidzein (98%) was purchased from Organic Technologies, Coshocton, OH. Dimethyl sulfoxide (DMSO; 99.9%), methanol (99.8%), trifluroacetic acid was purchased from ThermoFisher, Waltman, MA. Deionized (DI) water was used for the preparation of all diets.

### Insect colony

Neonate *T. ni* (Hübner) (Lepidoptera: Noctuidae) from a colony maintained on an artificial diet [General Purpose Lepidoptera Diet (GPLD), Frontier Agricultural Services, Newark, DE] at the USDA-ARS-National Center for Agricultural Utilization Research, Peoria, IL (NCAUR), were used for all bioassays. Standard protocol for preparing two liters of GPLD diet consisted of autoclaving 38 g of agar (Frontier Agricultural Sciences, Newark, DE) in 1.760 L of DI water at 250 °C for 20 minutes. The liquid agar was blended with 288 g of GPLD powder in a blender (HGB 100 Warring Blender, Dynamics Corporation of America, New Hartford, CT, USA) for approximately 5 minutes. Diet mixtures of GPLD were stirred by a IKA RW 20 digital stand mixer (IKA, Wilmington, NC) for approximately 2 minutes before dispensing about 3.3 mL of the molten diet treatment into each cup (Stock Number T125-0090, Dart Container Corporation, Mason, MI, USA). The insects used to start this colony were obtained from USDA-ARS, Biological Control Insect Research Laboratory, Columbia, MO before 1995.

### Baculovirus source

Stocks of the baculovirus provided by Thermo Trilogy (now Certis USA, Columbia, MD) were originally isolated from *A. falcifera* (Kirby) (Lepidoptera: Noctuidae), commonly known as celery looper. Subsequent baculovirus stock was produced *in vivo* at the USDA NCAUR via cabbage loopers following the modified procedures outlined in Behle *et al*.^11^. Briefly, AFMNPV was propagated by infecting third instar *T. ni* and harvesting larval corpses. Collected larvae were mixed with 0.5% SDS, homogenized, and filtered through cheese cloth. The filtrate was then centrifuged at 5000 rpm (5 °C) for 10 min, the supernatant was removed, and the pellet was resuspended in de-ionized water. Virus stock was then frozen for storage. The baculovirus stock utilized in this experiment was produced in May 2017 and contained 4.8 × 10^8^ occlusion bodies per milliliter (OB mL^−1^), determined microscopically using a hemacytometer. Baculovirus stock was frozen for long term storage and refrigerated at 5 °C for short term storage (up to 4 weeks).

### Plant growth

*Glycine max*: Soybean, (Proharvest A15U B146P), *Phaseolus vulgaris*: green bean (‘Savannah’), *Gossypium hirsutum*: cotton (‘DES 607’), and *Brassica oleracea*: cabbage (‘Bravo F1’) were grown in a greenhouse for approximately 5 weeks in 110 mm square plastic pots (Kord Products, Toronto, Canada). The potting mix used in the greenhouse was Redi-Earth (Sun Gro Horticulture, Bellevue, WA) fertilized with Osmocote Classic (14-14-14) and Micromax Micronutrients (The Scotts Company, Marysville, OH) per packaging recommendations.

### Spray equipment and parameters

Treatments were applied to plants with a research track sprayer (DeVries, Hollandale, MN) through a spray jet 80015 nozzle (Spray Systems, Wheaton, IL) programmed to deliver 36 mL of each treatment per pass at 179 kPa, equivalent to 340 L/Ha. The plants were positioned 50 cm below the nozzle and centered in the spray chamber.

### Insecticidal activity of AfMNPV applied to crops

#### Assay for insecticidal activity: leaf disk assay

Crop plants known to be hosts for *T. ni* (soybean, green bean, cotton and cabbage) were treated with AfMNPV in a dosage response assay. Five or more virus concentrations were prepared by 1:3 serial dilutions with water for application through the spray chamber as described above. Virus concentrations were mixed to provide application rates between 1.3 × 10^9^ and 1.0 × 10^12^ OB Ha^−1^. Rates applied to soybean required lower virus application rates compared with the other crops to in an effort to center the response near 50% larval mortality. After treatment, the spray residue was allowed to dry before five circular disks were cut from treated leaves for each crop by application rate combination. Leaf disks were placed individually in petri dishes with about 20 neonates, and sealed petri dishes were incubated in the dark at 28 °C for a 24 h feeding period. After feeding, six live larvae were randomly selected from each leaf disk and transferred to individual cups with GPLD for a total of 30 larvae crop × application rate combination. These insects were incubated for an additional six days, then evaluated for mortality. A control (no virus application) was included for each crop to assess handling mortality, which typically averaged less than 10%. Experiments were replicated three or more times on different days for a minimum of 90 larvae exposed for each crop × application rate combination. Insect mortality data were subjected to probit analysis (Polo Plus Software, LeOra Software LLC, CA) to determine LC_50_ values (Table 1). LC_50_ values were considered significantly different based on lethal dosage ratios in which the 95% confidence limits did not include a value of 1^12^.

**Table 1:** Insecticidal activity of AfMNPV against neonate *T. ni* feeding on treated plant leaf tissue. (LC_50_ and CL’s × 10^10^ OB/Ha).

Plant	LC_50_	Upper CL	Lower CL	Intercept	Slope	Heterogeneity	χ^2^	*df*	n
Soybean	1.77 a	2.19	1.39	−0.331	1.570	0.60	3.02	5	720
Cabbage	9.95 b	12.33	7.92	−0.603	1.521	0.74	2.98	4	510
Green bean	10.73 b	32.31	5.61	−0.418	1.034	0.15	0.45	3	450
Cotton	13.17 b	18.92	9.69	−0.679	1.592	1.53	4.59	3	599

#### Impact of leaf pubescence on insecticidal activity of AfMNPV

This experiment was conducted to evaluate the impact of leaf pubescence on the activity of the virus treatments applied to soybean plants by comparing shaved with un-shaved plants. Shaved plants were prepared by carefully removing the leaf pubescence from the upper leaf surface with a razor blade just prior to virus treatment. Following the techniques described above, greenhouse grown soybean plants (shaved and un-shaved) were treated with either a water control or a single LC_50_ rate of 1.8 × 10^10^ OB Ha^−1^ applied using the spray chamber. Neonates were allowed to feed on leaf disks excised from either control or virus treated leaves and evaluated for mortality as described above. The experiment was replicated three times. Differences between shaved and un-shaved treatments were compared using a paired-T test in SAS v9.4 (SAS Institute Inc., Cary, NC, USA).

### SEM analysis

Leaf tissue was excised, and sputter coated with Au particles: cotton, cabbage, green bean, and soybean (with and without trichomes) were analyzed. The coated leaf tissue was then examined using a JEOL 6400 V scanning electron microscope to evaluate differences in trichome density (JEOL USA Inc., Peabody, MA).

### Determining concentrations of flavonoids in soybean leaves

#### Sample preparation and extraction

Greenhouse grown plant leaf samples were freeze-dried and subsequently ground to a fine powder. Weighed samples of dried leaf powder (typically 0.25 g) were placed in a vial and extracted with 3 mL of a methanol:DMSO (1:1) solvent. The samples were typically sonicated for 30 minutes and allowed to stand overnight at room temperature. The extract was filtered through a 0.45 µM nylon 66 filter for HPLC analysis.

#### Analytical methodology

HPLC analysis was conducted following the protocol described in Dowd *et al*., 2018 and Riddick *et al*., 2018, on a Shimadzu LC-20 HPLC system (LC-20AT quaternary pump, DGU-20A5 degasser, SIL-20A HT autosampler, and a SPD M20A photodiode array detector, running under Shimadzu LCSolutions version 1.22 chromatography software, Columbia, MD, USA)^13,14^. The column used was an Inertsil ODS-3 reverse phase C-18 column (5 µ, 250 ×4.6 mm from Varian, Palo Alto, CA). For phenolic compound analysis, the initial conditions were 20% methanol with 0.25% trifluroacetic acid and 80% water with 0.25% trifluroacetic acid, at a flow rate of 1 mL min^−1^. The column was held at the initial conditions for 2 minutes, then developed to 100% methanol with 0.25% trifluroacetic acid in a linear gradient over 55 minutes. Five-point standard curves for the evaluation of the concentration of the identified flavonoids in soybean leaves were prepared with commercially purchased kaempferol, genistein, and daidzein for the determination of extinction coefficients at 280 and 340 nm.

#### LC-ESI-MS analysis for compound confirmation

Following the protocol described in Dowd *et al*., 2018 and Riddick *et al*., 2018, leaf extraction samples were run on an Thermo Electron LTQ Orbitrap Discovery Mass Spectrometer–a linear ion trap (LTQ XL) MS, coupled to a high precision electrostatic ion trap (Orbitrap) MS with a high energy collision (HCD) cell with an Ion Max electrospray ionization (ESI) source, and a Thermo Scientific ACCELA series HPLC system (ACCELA 1250 UHPLC pump, ACCELA1 HTC cool stack auto injector, and a ACCELA 80 Hz PDA detector) all running under Thermo Scientific Xcalibur 2.1.0.1140 LC-MS software (ThermoFisher Scientific, Waltham, MA)^13,14^. For phenolic analysis, the initial solvent system was 10% methanol verses water with 0.25% formic acid at a flow rate of 0.25 mL per minute. After injection the column was developed with a linear gradient to 100% methanol over 50 to 60 min. The software package was set to collect mass data between 100–2000 AMUs. Generally, the most significant sample ions generated under these conditions were [M-1]^−^ and [M + HCOO]^−^.

Six mass spec “events” were programed to run in sequence in the MS detection scheme. (1) LTQ(IT)-MS full scan m/z 150 to 2000. (2) LTQ(IT)-MS set to trap the most abundant ion above a threshold of 500 units and perform CID at 35% energy, with the resulting ions being detected by the IT-MS. 3) FT-MS (Orbitrap) full scan m/z 150 to 2000. (4) Mass-dependent MS/MS on the most abundant ion trapped by the IT-MS in Event 1 and perform HCD at 25% energy with the resulting fragmentation ions being detected by the FT-MS. (5) Mass-dependent MS3 on the most abundant fragment ion generated from Event 2 and perform HCD at 25% energy with the resulting fragmentation ions being detected by FT-MS. (6) Mass-dependent MS3 on the most abundant fragmentation ion generated from Event 2 and perform CID at 35% energy with the resulting ions being detected by IT-MS.

For the evaluation of Xcalibur accurate mass data by the Cerno BioScience LLC MassWorks 5.0.0.0 software the FTMS was set to collect spectra at a resolution of 7500 and a range of m/z of 100 to 2000 and then were evaluated by self-Calibrating Line-shape Isotope Profile Search, which enhances formula ID accuracy.

### Virus overlay bioassay of artificial insect diet with incorporated flavonoids

Artificial insect diet treatments containing genistein, daidzein and kaempferol were prepared to compare the effect of three flavonoids identified above on the insecticidal activity of the baculovirus. Treatments were prepared by incorporating the flavonoids into the insect diet using the same GPLD diet used for colony maintenance. Six diet treatments were prepared by mixing flavonoids in 100 mL insect diet at concentrations intended to approximate the dry weight concentrations of the flavonoids found in the soybean leaves (Table 2). Genistein (0.0029 g), kaempferol (0.0224 g), daidzein (0.0028 g), and a combination of the three flavonoids were dissolved in 500 µL of DMSO and incorporated into 100 mL of molten GPLD diet. The aglycone flavonoids are sparingly water soluble, and are only soluble in aprotic polar solvents, such as dimethylsulfoxide (DMSO)^15^. Two controls were used for the virus overlay experiment, a no additive control and a 500 µL DMSO solvent control. The no additive control treatment was made with an additional 500 µL of water. Prepared diet treatments were cooled at room temperature to solidify and stored in refrigeration at 5 °C until use.

**Table 2:** Concentrations of flavonoids found in fully expanded trifoliate soybean leaves (dry weight basis). Listed flavonoids were not detected in green bean, cabbage, or cotton leaves.

	(µM/g)	(µg/g)
Daidzein	0.678 ± 0.004	172 ± 1
Genistein	0.684 ± 0.009	185 ± 3
Kaempferol	4.832 ± 0.004	1382 ± 2

Insecticidal activity for the baculovirus in the presence of the flavonoids was evaluated by a dosage-response assay using a virus overlay exposure technique. Diet incorporated with genistein, kaempferol, daidzein, and a mixture of the three flavonoids (along with the DMSO and no-additive controls) were surface treated with 5 concentrations of baculovirus. Five concentrations of baculovirus were made by 1:3 serial dilutions to provide 3.3 × 10^4^ OB mL^−1^ to 4.1 × 10^2^ OB mL^−1^ concentrations. The stock virus preparation was counted under a microscope using a hemocytometer to verify the virus concentration. Each of the baculovirus concentrations plus a water control were applied to a set of 5 cups (= total of 30 cups) for each flavonoid incorporated diet preparation. Baculovirus treatments (0.1 mL per cup) were spread along the surface of the diet with a glass stir rod. After drying, approximately 12 *T. ni* neonates were added to each cup, and the cups were incubated for 24 hours in a dark incubator at 28 °C. To mimic the leaf assay above, larvae were allowed to feed for 24 hours, then 6 larvae from each cup were transferred individually to fresh cups containing GPLD, without the flavonoids, providing 30 insects exposed to each flavonoid × virus concentration. These larvae were incubated at 28 °C for 6 days, then evaluated for mortality. Each experiment was replicated at least 3 times on different days, using replicated diet preparations and different cohorts of insects. To determine whether flavonoid interactions were synergistic or additive the following equation was used with dosage response data (Eq. 1):

$$Cotoxicity\,factor=100\times \frac{(observed \% mortality-expected \% mortality)}{(expected \% mortality)}$$

Where a positive factor of ≥20 indicates synergistic potentiation, and a value of <20 to >0 indicates an additive potentiation^16–18^.

A second experiment, following the method stated above for virus overlay of incorporated diet, was conducted to evaluate the effect of higher concentrations of the individual flavonoids. The amounts of the flavonoids added to 100 mL GPLD were: 0.0186 g daidzein, 0.0836 g kaempferol, and 0.0186 g genistein. Experiments were repeated 3 times on different days as before.

## Results

**Figure 1:**
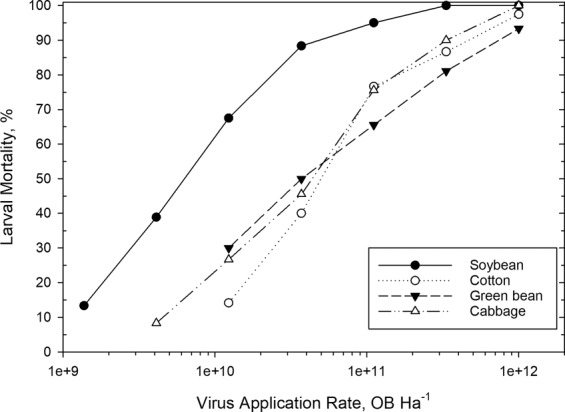
Insecticidal activity based on dosage-response of AfMNPV against *T. ni* neonates when applied to crop plants.

While investigating the effects of formulation ingredients to benefit the biopesticide AfMNPV against *T. ni* larvae^19^, virus LC_50_ rates were observed to vary widely across multiple plant species. Applications of AfMNPV were found to be more active when applied to soybean plants as compared to applications on cabbage, cotton, or green bean plants. The observed difference among these crop plants suggested that a component of the soybean plants may increase *T. ni* susceptibility to baculovirus or may enhance the baculovirus activity in some capacity. Thoroughly replicated experiments revealed that the *Af* baculovirus treatments were far more toxic to larvae that fed on treated soybeans as compared with the other three crops (Table 1, Fig. 1). The LC_50_ value for the virus applied to soybean was 1.77 × 10^10^ OB Ha^−1^, about six-fold greater toxicity when compared with virus applications to cabbage, green bean, or cotton. Toxicities among the latter three crops were not significantly different from one another based on lethal dosage ratios at the LC_50_ level. Mortality of larvae feeding on virus free plant leaves were less than 5% for all crops, with no significant differences observed in larval mortality or development.

This observed difference in activity among crop plants suggested a potential interaction between leaf morphology or chemical composition which was synergistic with *Af* baculovirus activity. Therefore, an investigation of the chemical differences in phenolic compound composition between soybean, green bean, cabbage and cotton crops was performed, which ultimately led to the identification of soybean exclusive flavonoids (within the tested species) genistein, kaempferol, and daidzein.

### Leaf pubescence

**Figure 2:**
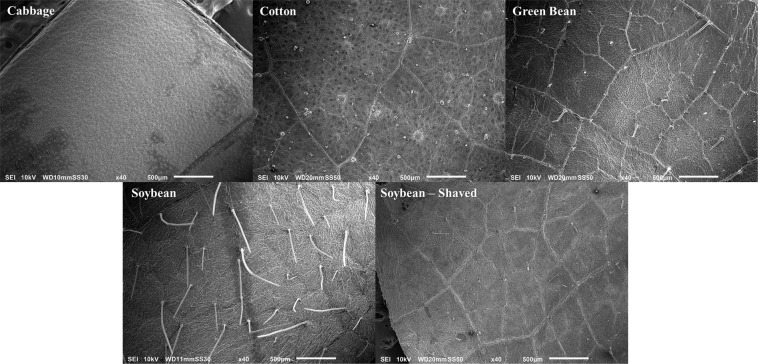
Scanning electron micrograph of cabbage, cotton, green bean, and soybean leaf tissue. In shaved soybean leaves, trichomes were carefully removed with a razor blade.

Each crop had differing degrees of leaf pubescence, cabbage lacking any and soybean having the most pubescence among the species tested (Fig. 2). To determine whether the physical morphology and structures of the leaf had any significant impact on virus activity, leaf trichomes were removed from soybean and virus activity was assessed. Removing pubescence from the leaf surface of soybeans did not significantly affect the insecticidal activity of the virus. Mortality of larvae exposed to virus applied to pubescent leaves averaged 64.6%, which was not significantly greater (mean difference = 6.3%, n = 6, SE = 9.2%, t = 0.69, pr > t = 0.52) than the mortality of larvae exposed to virus applied to leaves that were shaved to remove pubescence (58.3% mortality).

#### Leaf chemistry

Chemical analysis of leaves from the four crops grown under our experimental greenhouse conditions identified three flavonoid compounds from soybean that were not present in the other three crops. While the chemical differences between the plant species are likely to be extremely numerous, we focused on identifying significant differences in leaf phenolic composition as these are known to be produced as plant defense compounds. The identified flavonoid compounds were daidzein, genistein, and kaempferol and were present at concentrations of 0.17, 0.18, and 1.38 mg flavonoid g^−1^ dry leaf, respectively (Table 2). After identification, artificial diets were produced incorporating the flavonoid compounds, determining whether the flavonoid compounds enhanced virus activity and if the flavonoid phytochemicals were insecticidal on their own in virus free diet treatments.

#### Virus overlay of incorporated diet

Incorporating the three flavonoids into the artificial insect diet was intended to mimic the crop leaves and overlaying the surface of the diet with a virus treatment mimicked a spray application. Flavonoids were incorporated into the diet at the dry weight concentrations found in the soybean leaves. The virus free flavonoid diets did not cause a significant increase in larval mortality; all virus free control diets had <5% morality. Adding the flavonoids to the diet did significantly increase the insecticidal activity of the virus, but only for the combination treatment (Table 3). The LC_50_ value for the combined flavonoid treatment was significantly less than all other treatments; flavonoid combination synergistically improved baculovirus activity (co-toxicity factor approximately = 25). The highest LC_50_ value (lowest insecticidal activity) was measured for the GPLD diet control preparation, which was not significantly different from the DMSO or individual flavonoid treatments.

**Table 3:** Comparing insecticidal activity against neonate *T. ni* of AfMNPV applied as an overlay of insect diet with flavonoids incorporated. Significant differences among treatments compared based on lethal dosage ratio where the confidence limits do not include a value of 1.0. Daidzein, genistein and kaempferol were incorporated at rates approximating measured concentrations of soybean leaves. (LC_50_ and CL’s × OB/mL).

Diet incorporation	LC_50_	Upper CL	Lower CL	Intercept	Slope	Heterogeneity	χ^2^	*df*	n
GPLD only	3061 a	4050	2380	−5.975	1.714	1.31	17.06	13	450
Genistein	2736 a	3857	2028	−4.277	1.244	1.20	15.61	13	450
Daidzein	2638 a	4103	1796	−4.706	1.376	2.23	28.94	13	450
DMSO, surfactant	2363 a	2967	1912	−5.119	1.517	0.89	11.53	13	451
Kaempferol	2307 a	2884	1862	−5.280	1.570	0.89	11.56	13	449
Combination	1666 b	2317	1217	−4.890	1.518	1.82	23.60	13	450

Incorporating higher concentrations of genistein, daidzein, and kaempferol into insect diet improved the insecticidal activity of the virus overlay treatment (Table 4). The high concentration of kaempferol (estimated at 3.6 × greater than leaf concentrations) improved the insecticidal activity (LC_50_ = 628 OB mL^−1^) of the virus 4.2 × compared with diet alone (LC_50_ = 2668 OB mL^−1^) or 3.9 × DMSO diet controls (LC_50_ = 2431 OB mL^−1^). The high concentration of genistein (estimated at 6.5 × greater than leaf concentrations) improved virus potency about 2.3 × compared to the control diet, with an LC_50_ at 1196 OB mL^−1^. The high concentration of daidzein (estimated at 6.5 × greater than leaf concentrations) improved the insecticidal activity of the virus 1.5 × compared to the control diet, with an LC_50_ at 1757 OB mL^−1^. High doses of daidzein, kaempferol, and genistein were found to synergistically improve virus activity, with co-toxicity factors of approximately 20, 60, and 150, respectively. The virus free high concentration flavonoid diets did not cause a significant increase in larval mortality; all virus free control diets had <5% morality.

**Table 4:** Comparing insecticidal activity against neonate *T. ni* of AfMNPV applied as an overlay of insect diet with high doses of flavonoids incorporated into the diet. Significant differences among treatments compared based on lethal dosage ratio where the confidence limits do not include a value of 1.0. Daidzein, genistein and kaempferol were incorporated at rates 6.5, 6.5, and 3.5 times their approximate concentration in soybean leaves, respectively. (LC_50_ and CL’s × OB/mL).

Diet incorporation	LC_50_	Upper CL	Lower CL	Intercept	Slope	Heterogeneity	χ^2^	*df*	*n*
GPLD only	2669 a	5051	1148	−5.167	1.508	6.25	81.27	13	445
DMSO, surfactant	2431 a	4224	1197	−5.024	1.484	4.77	61.97	13	443
Daidzein 6.5x	1758 b	2140	1451	−5.627	1.734	0.93	2.779	3	450
Genistein 6.5x	1196 b	2466	299	−4.368	1.419	7.19	93.40	13	441
Kaempferol 3.5x	628 c	1213	140	−4.019	1.436	4.91	63.77	13	445

## Discussion

AfMNPV treated soybean had, on average, a 6-fold increase in virus activity compared with the other crops. Three flavonoid compounds (daidzein, genistein, and kaempferol) were uniquely found only in the soybean crop, and were not detected in cotton, cabbage, or green bean plant matter. The soybean flavonoid compounds were found to synergistically improve baculovirus activity against *T. ni*. Of the four crop species tested, soybean not only enhanced viral activity, but also had the greatest amount of leaf pubescence (trichomes); cabbage was entirely glabrous, or lacking trichomes (Fig. 2). Leaf pubescence in soybean provides insect resistance to lepidopteran larvae^20^. Larval stage defoliating insects show diminished fitness with reduced growth and delayed development on soybean with normal or dense pubescence when compared with glabrous soybean lines^21^. Leaf trichome orientation and tip morphology have a significant impact on soybean insect resistance, with sharper and more perpendicular trichomes (in relation to the leaf surface) increasing resistance to insect attack. For *T. ni*, it had been previously observed that the presence of trichomes on soybean leaves decreased feeding and increased mortality with trichome density; the removal of the trichomes on the youngest fully expanded leaves resulted in a significant increase in *T. ni* feeding^22^. In this experiment, *T. ni* feeding on the youngest fully expanded soybean leaves did not display any increased mortality when compared between shaved and unshaved leaves without a virus treatment (all virus-free treatments <5% mortality). Additionally, there was no significant differences in viral activity (mortality) in the *T. ni* fed on shaved vs. unshaved leaves that were treated with virus (pr > t = 0.52). Therefore, the trichomes of soybean do not play a significant role in the increased activity of AfMNPV against *T. ni*.

After determining trichomes did not affect virus activity, the chemical composition of each crop was analyzed particularly looking for differences in flavonoid concentration. Flavonoid compounds are known to be involved in numerous biochemical processes including defense functions against insect pests^23^. As such, analytical efforts concentrated on defining the amount and types of flavonoids present in soybean and either not present, or to a reduced extent, in the other crops. When comparing the flavonoid composition of the four crop species, three flavonoid compounds were produced only by soybean: genistein, kaempferol, and daidzein.

The isoflavonoids genistein and daidzein are typically abundant in soybean and other legumes and are of great dietary interest as bioactive agents^24–26^. Kaempferol is a flavonoid which falls into the subcategory of flavonol, and is also found to be abundant in soybean leaves^27^. However, green bean (*Phaseolus vulgaris* L.) had no detectible genistein, kaempferol, or daidzein, despite being a legume in the same family as soybean (Fabaceae). Isoflavonoids are often produced by plants in response to biotic stress and can be utilized to inhibit pathogen growth and help protect plant tissues from insect herbivory^28–30^. Flavonoids play a highly complex role in plant-insect interactions that goes beyond simple broad spectrum defense mechanisms and are often highly species specific^23^. Furthermore, these compounds are involved in a myriad of biochemical functions including inducers of nodule formation and symbiosis between soybean roots and bacteria^31^.

As these compounds were only found in soybean, each was selected and incorporated into artificial diet. At leaf level concentrations, none of these compounds interacted with the virus treatment to improve insecticidal activity. However, the combination treatment had a significantly greater virus activity. This observation may have been the result of the overall increase in flavonoid content in the combination treatment. Experiments using higher concentrations of the genistein and kaempferol also improved virus activity, supporting the theory that the observed affect was concentration dependent.

**Figure 3:**
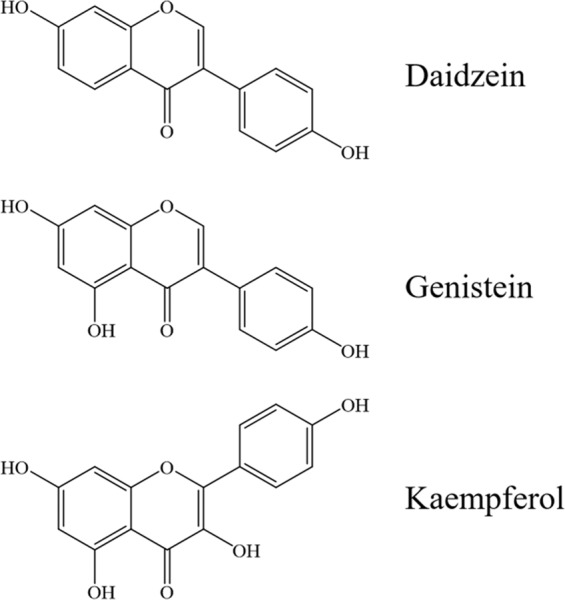
Chemical structures of flavonoid compounds.

Genistein, kaempferol, and daidzein all originate from a Shikimate pathway derived phenylalanine, and contain two phenyl rings and a heterocyclic ring^32^. Genistein and daidzein vary only in their number of hydroxyl groups, while kaempferol has additional hydroxyls and the second phenyl ring attached at the ether carbon of the heterocyclic ring (Fig. 3). All three of the identified flavonoids are known anti-oxidants, with the aglycone genistein having the highest Trolox Equivalent Antioxidant Capacity (2.90 mM), compared with kaempferol (1.34 mM) and daidzein (1.26 mM)^33^. This may help explain the differences observed in the viral lethality, since the prevention of the inflammatory response and quenching of reactive oxygen species would inhibit apoptosis of infected cells. Reactive oxygen species are typically produced as an antimicrobial response to infection, and are involved in the induction of apoptosis and inflammation^34,35^. The relatively simplistic insect immune system relies on the rapid apoptosis of baculovirus infected cells to terminate virus multiplication by premature cell lysis^36,37^. The early onset of apoptosis in infected insect cells can dramatically reduce virus concentrations, with reductions up to 50 fold^37,38^. Oxidative stress in the midgut of lepidopterans due to plant-mediated peroxidase activity has been shown to reduce baculovirus activity and insect death^39^. The rapid sloughing of infected midgut epithelial cells before the establishment of a systemic infection reduces viral susceptibility, and reactive oxygen species may promote sloughing by damaging midgut cells^40^. A reduction or delay in apoptosis or sloughing of infected insect cells could increase the viral titer released in the insect, improving the chance for lethal infection, and leading to a greater degree of mortality.

Estrogen (7β-estradiol) has been observed to be anti-apoptotic, inhibiting reactive oxygen species production, chronic inflammation, auto-aggressive immune responses, and cell apoptosis (except in osteoclasts) in mammals^41^. Genistein is one of the most potent phytoestrogenic flavonoids, with much greater binding affinity to estrogen receptors than either kaempferol or daidzein^42^. Lepidoptera which were fed a diet containing estrogen showed decreased larval weight and delays in development, though no significant difference was observed with regard to insect mortality^43^. This is consistent with the diet overlay study and suggests the phytoestergenic compounds themselves do not cause mortality in the insects but may interact with the baculovirus by inhibiting the inflammatory and apoptotic cell responses. This could increase the rate of viral production in the *T. ni* and result in greater insect mortality.

The combined leaf level concentrations of flavonoids had a significantly increased lethal dose response (approximately 2-fold), but only partially accounts for the 6-fold increase in virus activity observed for soybean. Increasing the concentrations of the flavonoids genistein and kaempferol well above the physiological concentrations in the artificial diet resulted in increased virus activity. Importantly, even at the highest concentrations of flavonoid incorporation there was no increase in larval mortality in the no-virus controls. Therefore, the flavonoids are not acting as broad-spectrum defense compounds against the *T. ni*. Genistein and kaempferol did increase virus activity (2.3–4.2 ×) at higher concentrations of the individual flavonoid compounds and had a synergistic interaction with the baculovirus. The smaller increase in virus activity observed in the daidzein diets (1.5 ×) may be due to daidzein having the lowest anti-oxidant and phytoestergenic activity, as compared with genistein and kaempferol^33,42^. Daidzein can often produce distinctly different physiological and cell signaling responses despite a minor structural difference with genistein^44^.

Further studies are underway to compare various soybean cultivars and identify potential genotypic variability. Comparisons will be performed to further elucidate the soybean components responsible for the synergistic increase in baculovirus activity. The significant interactions between plant flavonoids, entomopathogenic baculovirus, and increased insecticidal activity suggests a potential plant breeding objective to improve plant insect resistance concurrent with an integrated pest management system.
